# Inhibition Mechanism of *Cinnamomum burmannii* Leaf Essential Oil Against *Aspergillus flavus* and Aflatoxins

**DOI:** 10.3390/foods14040682

**Published:** 2025-02-17

**Authors:** Huanyan Liang, Feifei Lv, Mengting Xian, Chenghua Luo, Lei Zhang, Meihua Yang, Qian Li, Xiangsheng Zhao

**Affiliations:** 1 Institute of Medicinal Plant Development, Chinese Academy of Medical Sciences & Peking Union Medical College, Beijing 100193, China; liang448594255@163.com (H.L.); yangmeihua15@hotmail.com (M.Y.); 2 Hainan Provincial Key Laboratory of Resources Conservation and Development of Southern Medicine, Hainan Branch of the Institute of Medicinal Plant Development, Chinese Academy of Medical Sciences & Peking Union Medical College, Haikou 570311, China; lvfei.fei1988@163.com (F.L.); 18898966081@163.com (M.X.); luochenghua30@163.com (C.L.); 3 School of Chinese Materia Medica, Guangdong Pharmaceutical University, Guangzhou 510006, China; zhanglei-ctcm@gdpu.edu.cn; 4 College of Basic Medical Science, Ningxia Medical University, Yinchuan 750004, China

**Keywords:** antifungal, aflatoxin B_1_, mode of action, in vivo efficacy, biofumigant

## Abstract

This investigation evaluates the comparative efficacy of *Cinnamomum burmannii* leaf essential oil (YXYO) and its main active ingredients as a novel preservative to protect stored food commodities from fungal infestations, aflatoxin B_1_ (AFB_1_) contamination caused by *Aspergillus flavus*. Morphological observations utilizing SEM and TEM revealed significant alterations in treated samples, alongside a decrease in ergosterol content and a dose-dependent disruption of the antioxidant system and energy system. Transcriptomic analysis suggested that differentially expressed genes were predominantly associated with spore growth, the cell wall, the cell membrane, oxidative stress, energy metabolism, and aflatoxin biosynthesis. Solid-phase microextraction–gas chromatography–mass spectrometry (SPME-GC-MS) identified ten active ingredients in YXYO, including borneol, *α*-terpineol, terpinen-4-ol, etc. Moreover, an effective inhibition of *A. flavus* infection in peanuts was observed with the application of 30 μL/disc of YXYO and a blend of its active compounds.

## 1. Introduction

*Aspergillus flavus* is a ubiquitous filamentous fungus that can colonize agricultural commodities like tree nuts, peanuts, maize, rice, and herbs during preharvest, harvest, storage, and transport, leading to the production of several mycotoxins [1]. Aflatoxins, the secondary metabolites mainly produced by *A. flavus*, have previously been found to induce various toxic effects such as carcinogenicity, teratogenicity, mutagenicity, and immunotoxicity [2]. Up to now, more than 20 different types of aflatoxins have been discovered; of these, aflatoxin B_1_ (AFB_1_) is recognized as the most toxic and classified as a group I carcinogen by the International Agency for Research on Cancer [3]. As a result, numerous countries and international organizations have defined the maximum aflatoxin limit in their agricultural commodities. For example, the limit for AFB_1_ in maize, peanuts, and their derived products is less than 20 ug/kg [4]. Furthermore, according to the 2020 edition of the Chinese Pharmacopeia, the permissible upper limits for aflatoxins in various herbs are established at 5 µg/kg for AFB_1_ and a total of 10 µg/kg for the combined presence of AFB_1_, AFB_2_, AFG_1_, and AFG_2_. The widespread occurrence of fungi and the subsequent contamination by aflatoxins result in substantial economic losses and pose significant public health challenges worldwide. Therefore, it is urgent to implement effective measures for the prevention and control of *A. flavus* growth and aflatoxin production in agricultural commodities.

Currently, different control strategies have been developed to reduce fungal contamination and mycotoxin synthesis in agricultural products, including chemical, physical, and biological methods [5]. Among them, physical approaches (including irradiation, thermal, ozone, etc.) are associated with safety concerns and cannot always be feasibly applied [6]. Biological prevention strategies are implemented utilizing specific microorganisms (yeasts, bacteria, nontoxigenic fungi, etc.) to mitigate aflatoxin contamination. The negative effects of this technology stem from competition for space and nutrients and biological interactions like antibiosis [7]. Chemical agents, such as salts, acids, oxidizing agents, and alkaline, have been proved to be effective for the control of *A. flavus* contamination, with a low cost and convenient application compared with physical and biological methods [8]. Unfortunately, the application of fungicides can result in environmental pollution and pose health risks [9]. Therefore, there is an urgent need to find effective and nontoxic natural antifungal agents to improve safety.

From the viewpoint of agricultural product safety, essential oils from plants and herbs are considered to have the most promising market potential for inhibiting fungal growth and limiting aflatoxin production based on the characteristics of facile volatilization, low toxicity, superior biodegradation, and environmental friendliness [2]. Recent studies have demonstrated that certain essential oils can effectively inhibit *A. flavus* growth and aflatoxin contamination, such as *Cinnamomum cassia* [10], *Cymbopogon martinii* [2], *Ocimum americanum* [11], etc. *Cinnamomum burmannii* (Lauraceae family) is an evergreen tree predominantly found in Indonesia, Vietnam, the Philippines, and China [12]. The bark of *C. burmannii* is commonly used in traditional medicinal practices and functions as a spice and flavoring agent [13]. The leaves of *C. burmannii* exhibit significant antioxidant and antimicrobial properties and have been utilized as food additives [14,15]. In our previous research, we found that the essential oil of *C. burmannii* leaves (commonly referred to as Yinxiang Ye in Chinese) can significantly inhibit the growth of *A. flavus* and the accumulation of aflatoxins [16]. However, the inhibitory mechanism of *C. burmannii* leaf essential oil against *A. flavus* has not been reported.

Therefore, this study was conducted to explore the antifungal mechanism against *A. flavus* and the main active composition of *C. burmannii* leaf essential oil (YXYO). The morphological, physiological, and biochemical alterations in *A. flavus* after YXYO treatment were examined. Concurrently, transcriptomic analysis was used to identify the differentially expressed genes in *A. flavus* exposed to YXYO. Furthermore, the components of YXYO were characterized by SPME-GC-MS, and the antifungal effects of the main compounds were assessed. Finally, the application of YXYO to inhibit *A. flavus* infection in peanuts was demonstrated. This work will be conducive to inspiring future investigation on aflatoxin contamination control in food and agricultural products.

## 2. Materials and Methods

### 2.1. Strains, Materials, and Chemicals

The *A. flavus* strain (CGMCC 3. 4410) was obtained from the General Microbiological Culture Collection Center (Beijing, China). The strains were kept on potato dextrose agar (PDA) medium (1 L of distilled water contains 40 g of Solarbio-PDA). The standard solutions of AFB_1_ and [^13^C_17_]-AFB_1_ were purchased from Pribolab (Singapore). HPLC-grade acetonitrile, methanol, formic acid, acetic acid, and ammonium formate were bought from Merch (Darmstadt, Germany). Ultrapure water was prepared by the Milli-Q system (Millipore Corporation, Bedford, MA, USA). The standard of ergosterol was purchased from Shanghai Yuanye Bio-Technology Co., Ltd. (Shanghai, China). Sabinene, *β*-myrcene, bornyl acetate, *α*-terpineol, terpinen-4-ol, borneol, terpinolene, *p*-Cymene, *α*-phellandrene, and eucalyptol were bought from Shanghai Aladdin Biochemical Technology Co., Ltd. (Shanghai, China).

The leaves of *C. burmannii* were collected from Haikou (Hainan province, China) and identified by Professor Xinquan Yang (Hainan Branch of the Institute of Medicinal Plant Development, Chinese Academy of Medical Sciences and Peking Union Medical College). The fresh leaves were chopped and extracted by hydrodistillation. The essential oil was dehydrated using anhydrous sodium sulfate (Na_2_SO_4_) and stored at 4 °C.

### 2.2. Culture and Conditions

Aliquots (2 μL) of spore suspension (10^6^ spores/mL) were spot-inoculated to the center of a Petri dish (90 mm diameter) containing PDA medium. Then, different concentrations of YXYO were added to sterile filter papers which were taped to the center of the inner surface of the plate cover. These plates were sealed with parafilm (Bemis, Neenah, WI, USA) and incubated for 7 days at 28 ± 2 °C. Samples without YXYO treatment were regarded as the control (CK).

### 2.3. Determination of YXYO Effect on the Growth of A. flavus

The antifungal activity of YYXO was determined in in vitro conditions, for the assessment of the volatile phase effect on the mycelial growth of *A. flavus*. The methodology used was an adaptation of C. Soares et al. [17]. The diameters of the growing colonies were measured daily till day 7. For each dose, three replicate plates were used. The percentage of mycelial growth inhibition was calculated using the following formula: when the mycelium of the negative control group spread to the edge of the Petri dish, the colony diameter of the control group (*D*_0_) and treated group (*D_t_*) was measured, and the growth inhibition rate (*IR*) towards *A. flavus* was calculated according to the following Equation (1):

(1) $$IR\left(\%\right)=\left(D_{0}-D_{t}\right)/D_{0}\times 100\%$$

The mycelial weights were measured after incubation. The medium was heated, and the mycelia of all groups were filtered through Whatman filter no. 1 and then washed with distilled water. The mycelia were placed on preweighed Petri plates and were allowed to dry at 80 °C for 6 h and then at 40 °C overnight. The percent growth inhibition on the basis of dry weight was calculated using the following formula: the weights of the control group (*W*_0_) and treated group (*W_t_*) were measured, and the growth inhibition rate (*IR*) towards *A. flavus* was calculated according to the following Equation (2):

(2) $$IR\left(\%\right)=\left(W_{0}-W_{t}\right)/W_{t}\times 100\%$$

### 2.4. Scanning (SEM) and Transmission (TEM) Electron Microscopy

*A. flavus* mycelia (three days old) exposed to YXYO (0, 10, 20, and 30 μL/disc) for four days were observed by SEM. The segments were washed according to Q. Gong et al. [18]. Briefly, the conidia were fixed in 2.5% glutaraldehyde (prepared in 0.1 M sodium phosphate buffer) at 4 °C for 24 h, rinsed 3 times with phosphate buffer (0.02 M), and subsequently fixed with 2% osmium tetroxide for 2 h at 20 °C and dehydrated with an ascending concentration of ethanol (30, 50, 70, 80, 90, 100%) for 10 min each, CO_2_-critical-point-dried, and sputter-coated with gold. Samples were kept in a desiccator until examination with a scanning electron microscope (Hitachi SU8100, Tokyo, Japan).

Fresh mycelia were fixed with 2.5% (*v*/*v*) glutaraldehyde and 4% (*v*/*v*) paraformaldehyde at 4 °C for 4 h. After dehydration, ultrathin section preparation, and staining, mitochondria inside mycelia were visualized with a transmission electron microscope (JEM-1400, JEOL Ltd., Tokyo, Japan), and images were displayed with SightX viewer (JEOL Ltd., Tokyo, Japan).

### 2.5. Effect of YXYO on Oxidative Impairment and Energy Metabolism

#### 2.5.1. Reactive Oxygen Species (ROS) Production and Antioxidant-Related Enzyme Assays

The hyphae of *A. flavus* (three days old) which were incubated in PDA with or without YXYO treatment for 12 h were washed with 0.01% PBS solution. The production of intracellular ROS was tested using 2′,7′-dichlorofluorescein diacetate (DCFH-DA) for 30 min at 37 °C in the dark. Then, this was observed and photographed under a laser confocal fluorescence microscope (Leica TCS SP8, Wetzlar, Germany).

The mycelia of *A. flavus* (three days old) were treated with different concentrations of YXYO and cultured in an incubator shaker (140 rpm, 25 °C). After incubation, the mixture was centrifuged (8000× *g* and 4 °C) for 10 min to remove the supernatant. Then, it was preserved at −80 °C for enzyme activity detection. The enzyme activities of catalase (CAT), glutathione peroxidase (GPx), superoxide dismutase (SOD), and malondialdehyde (MDA) were measured using commercial kits (Nanjing Jiancheng Bioengineering Institute, Nanjing, Jiangsu, China).

#### 2.5.2. Detection of ATPase, Succinate Dehydrogenase (SDH), and Malic Dehydrogenase (MDH) Activity

The pretreatment procedure is identical to that utilized for the assessment of antioxidant-related enzymes. The ATPase activity and SDH and MDH enzyme activity of *A. flavus* mycelia were determined using commercial kits (Nanjing Jiancheng Bioengineering Institute, Nanjing, Jiangsu, China). All steps were carried out according to the kit instructions. Enzyme activities were expressed as units per milligram of protein.

### 2.6. Transcriptome Analysis

The mycelia of *A. flavus* were collected after incubation with 0 and 20 μL/disc of YXYO for 7 d for transcriptomic analysis. The mycelial samples were immediately and rapidly frozen in liquid nitrogen and then stored at −80 °C. There were three biological replicates for each sample. The total RNA was extracted using the TRIzol reagent. The RNA Nano 6000 Assay Kit of the Bioanalyzer 2100 system (Agilent Technologies, Santa Clara, CA, USA) was used to assess RNA integrity and gross amount. The total RNA was then purified by poly-Toligo-attached magnetic beads and broken into short fragments. The random hexamers and dNTPs were used to synthesize the first-strand and second-strand cDNA, respectively. The cDNA library was constructed by PCR amplification after purification, size fractionation (AMPure XP beads), and Illumina adapter ligation. An Agilent 2100 Bioanalyzer was applied for the quality control of the cDNA library. All 9 libraries were sequenced on the Illumina HiSeq platform, and 150 bp paired-end reads were generated.

The raw reads of low quality and reads containing adapter and ploy-N were removed by SMRTlink 5.1 software to obtain high-quality clean reads. The high-quality paired-end clean reads were aligned to the *A. flavus* reference genome from NCBI (http://www.ncbi.nlm.nih.gov (accessed on 15 August 2020)) using HISAT2 v2.0.5. In a reference-based approach, the mapped reads of each sample were assembled by StringTie (v1.3.3b). The gene expression levels were calculated by the fragments per kilobase of transcript per million reads (FPKMs) which is based on the length of the gene and read count mapped to genes. The differentially expressed genes (DEGs) were detected by the DESeq2R package (1.20.0) based on genes with an adjusted *p* value < 0.05 and |log2 (fold change)| > 1. Functional enrichment analysis, including Kyoto Encyclopedia of Genes and Genomes (KEGG) and Gene Ontology (GO) enrichment analysis, was implemented by KOBAS v2.1.1 and Goatools v0.6.5 software.

### 2.7. Quantification of Aflatoxin B_1_ and Ergosterol

The content of AFB_1_ and ergosterol was determined using ultra-high-performance liquid chromatography coupled to tandem quadrupole mass spectrometry (UPLC-MS/MS, XEVO-TQ, Waters, Milford, MA, USA). The detection and qualitative analysis were operated in positive electrospray ionization (EI) mode with MRM scanning mode. The chromatographic and mass spectrometry conditions for aflatoxin B_1_ detection were consistent with our previous research [19]. Ergosterol was separated on a Waters C_18_ column (100 mm × 2.1 mm, 1.8 µm) with the room temperature set at 30 °C. Methanol (A) and 0.1% aqueous formic acid (B) were used as the mobile phase. The flow rate was set at 0.25 mL/min. The gradient procedure was as follows: start with 90% A; increase to 98% A from 0 to 5 min; and decrease to 90% A from 5 to 8 min. The transitions of ergosterol for quantification and identification were 379.60 > 68.9 and 379.6 > 124.9, respectively.

The sample preparation for aflatoxin was performed according to our previous method [16]. The extraction of ergosterol from *A. flavus* was conducted according to the method of L. He et al. [20], with slight modifications. Briefly, 0.05 g of powdered *A. flavus* mycelium was accurately weighted and loaded into a 5 mL centrifuge tube. The ergosterol was extracted by 3 mL MeOH in an ultrasonic bath for 30 min (25 °C, 40 Hz, 50 W) and centrifuged at 13,000 rpm for 2 min. Subsequently, the extracted solution was filtered with a 0.22 μm microporous membrane before injection into the UPLC-MS/MS system.

### 2.8. Analysis of the Volatile Components of YXYO

Solid-phase microextraction–gas chromatography–mass spectrometry (SPME-GC-MS) was used to analyze the volatile compounds in YXYO. The SPME fiber (carboxen/polydimethylsiloxane/divinylbenzene, 50 mm, Bellefonte, PA, USA) was conditioned at 260 °C for 60 min. Extraction was carried out by placing YXYO (1 μL) in a 15 mL amber vial to adsorb volatile compounds at 30 °C for 5 min and then immediately injecting it into the GC injection port at 250 °C for 3 min to desorb volatile compounds. Volatile compounds were analyzed using a gas chromatograph (Agilent 6890A, Santa Clara, CA, USA) equipped with a 5975C quadrupole mass spectrometer on a DB-5MS capillary column (30 m × 0.25 mm, 0.25 μm, Agilent Technologies, Santa Clara, CA, USA). The carrier gas was helium at a constant flow rate of 1.0 mL/min. The temperature program used was as follows: initial maintenance at 50 °C, increase to 145 °C at 3 °C/min, and increase to 250 °C at 1 °C/min. The mass-selective detector was operated with an electron energy of 70 eV in electron ionization (EI) mode. The injection, transfer line, and ion source temperatures were 250 °C, 250 °C, and 150 °C, respectively. The MS data were obtained in full-scan mode (*m*/*z* 40~550 amu). The volatile compounds were identified by using standards, comparing their mass spectra to those of compounds in the National Institute of Standards and Technology (NIST) and retention indices (RIs). The RIs of the compounds were calculated using a homologous series of n-alkanes (C_7_–C_40_; Sigma-Aldrich, St. Louis, MO, USA).

### 2.9. In Vivo Efficacy of YXYO on A. flavus Infection in Peanuts

The antifungal effect of YXYO was tested using the fumigation method. Briefly, 10 g of the UV-irradiated peanuts was placed in a Petri dish (90 mm diameter) and infected with 10 μL of spore suspension (10^6^ spores/mL). Then, 30 μL of YXYO and a blend of its active ingredients was added to sterile filter papers which were taped to the center of the inner surface of the plate cover. These plates were sealed with parafilm (Bemis, Neenah, WI, USA) and incubated for 7 days at 28 ± 2 °C. The AFB_1_ in infected peanuts was determined using the LC-MS/MS method.

### 2.10. Statistical Analysis

All experiments were conducted in duplicate. The results were assessed for significant differences (*p* < 0.05) and then subjected to one-way analysis of variance using the IBM SPSS statistical software 23.0 (IBM Corporation, Armonk, NY, USA).

## 3. Results and Discussion

### 3.1. In Vitro Evaluation of Antifungal and Antiaflatoxin Activity of YXYO Against A. flavus

**Figure 1:**
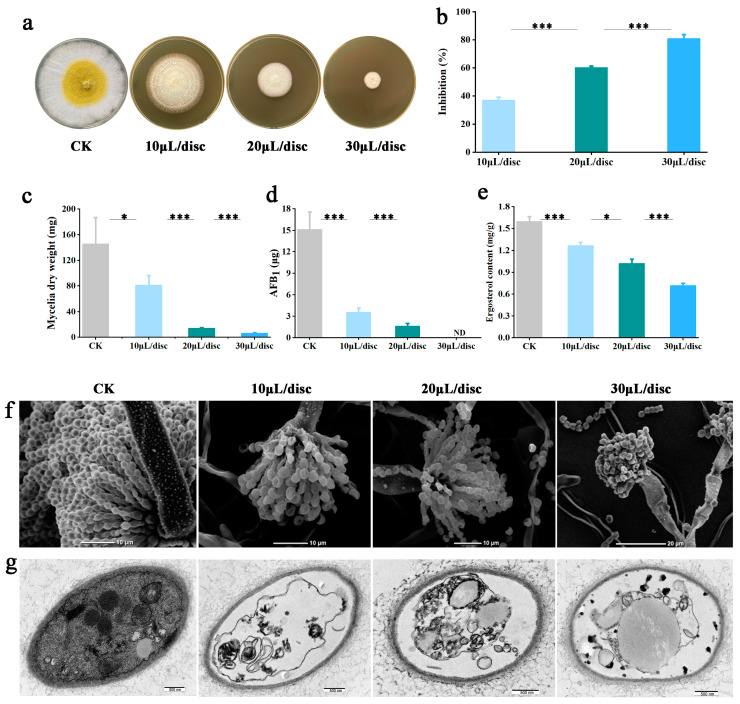
In vitro inhibitory effects of YXYO on (**a**) *A. flavus* growth, (**b**) colony diameter, (**c**) mycelium dry weight, (**d**) AFB_1_ content, and (**e**) ergosterol content; (**f**) SEM image of *A. flavus* conidiophores; and (**g**) TEM image of *A. flavus* cell. (*, *p* < 0.05; ***, *p* < 0.01).

To define the inhibitory effect of YXYO on the development and toxigenicity of *A. flavus*, the fungal colony diameter, mycelial dry weight, and aflatoxin production under different treatments in PDA plates for 7 days were quantified. As shown in Figure 1, YXYO exerted inhibitory effects on the mycelial growth, mycelial dry weight, and AFB_1_ content at the tested concentrations. The colony diameter inhibition increased significantly (from 36.96% to 80.94%) with increasing YXYO concentration (10, 20, 30 μL/disc) (Figure 1b). Similarly, the dry weight of the mycelia showed an evident reduction with increasing YXYO concentrations (Figure 1c). Notably, the LC-MS/MS determination results indicated that YXYO significantly inhibited AFB_1_ production. The content of AFB_1_ was 15.12 μg in CK, whereas no AFB_1_ was detected when the concentration of YXYO was raised to 30 μL/disc (Figure 1d). It is noteworthy that the application of 30 μL/disc of YXYO did not entirely suppress the growth of *A. flavus* mycelium; however, it was effective in completely inhibiting the production of aflatoxin. Similar results were obtained by S. Das et al. [21], and J. V. Gómez et al. [22]. In our previous study, it was found that following a 7-day treatment with 30 μL/disc YXYO, an additional 26 days were required for the diameter of *A. flavus* colonies to reach the same size as that of the control group, and the AFB_1_ production rate experienced significant inhibition [16]. The results of the antifungal and antiaflatoxin ability test indicated that YXYO displays strong inhibitory effects on the fungal development and aflatoxin synthesis of *A. flavus*. To investigate the antifungal and antiaflatoxin mechanisms of YXYO, three concentrations were selected for subsequent experiments.

### 3.2. Effect of YXYO on Morphology and Ultrastructure of A. flavus

The antifungal effects of YXYO might be attributed in part to substantial damage to the mycelium cell structure and organelles. The morphological and ultrastructure alterations in *A. flavus* were observed using electron microscopy to investigate the antifungal mode of YXYO.

According to the SEM observation of the control set, the intact morphology was normal and regular, the surface of the mycelium was smooth and full, and the conidia could be clearly observed (Figure 1f). When *A. flavus* was treated with YXYO, the morphological characteristics of the mycelium showed notable alterations compared to the control group. The morphology of the mycelium was aggregated, shrunken, and distorted. With increasing concentrations of YXYO, the deformation in the mycelium morphology was increasingly pronounced. Additionally, the spores of the control group were normal and intact, whereas those treated with YXYO exhibited surface wrinkles and showed signs of breakage. These results were similar with the phenotypic variations in *A. flavus* treated with other plant extracts, such as *Illicium verum* Hook.f. essential oil [23], *Cinnamomum zeylanicum* Blume essential oil [24], and Cuminaldehyde as the predominant component in cumin essential oil [25]. The observed distortion in the treated cells can be attributed to the leakage of cellular contents, which was further corroborated by TEM observations.

In order to explore the effect of YXYO on cell ultrastructure, TEM was used (Figure 1g). The TEM of untreated *A. flavus* revealed a normal state with integrated internal and external tissue structure, a uniform distribution of organelles, and a complete and continuous cell wall. In the treated samples, the *A. flavus* mycelium cell showed the morphological characteristics of contraction and plasmolysis, the distribution of mitochondria was sparse, and there was slight shrinkage. When the concentrations of YXYO were further increased, a large area of vesicles appeared in the mycelial cells, the internal composition of the cells was disordered, the mitochondria were obviously shrunk, seriously damaged, and the content of the cytoplasm decreased. There was no significant damage to the cell wall observed, consistent with previous investigations of A. Kedia et al. [26]. However, the cellular destruction induced by YYXO in this study was comparable to the effects of paeonol on *Aspergillus flavus*, which demonstrated degenerative alterations in the cell wall [27]. Therefore, it may be concluded that the plasma membrane serves as a principal target site for YYEO, as has been previously emphasized for antifungal essential oils and their main components [28].

### 3.3. Effect of YXYO on Ergosterol Content

The cell membrane plays important roles in the maintenance of osmotic pressure and normal function. Ergosterol is an important and specific component of the fungal cell membrane, and it is essential for fungal growth and development. Ergosterol is considered to be crucial in regulating cell membrane fluidity, permeability, and membrane-bound enzyme activities, as well as in substance transportation [29]. Therefore, many antifungal agents inhibit fungal growth by interrupting ergosterol biosynthesis [30,31]. The effect of YXYO on the ergosterol content of *A. flavus* was determined under treatment conditions of 10 μL/disc, 20 μL/disc, and 30 μL/disc. The dose-dependent decrease in the ergosterol content corresponded to the increasing concentrations of YXYO (Figure 1e). The ergosterol production of the 10 μL/disc, 20 μL/disc, and 30 μL/disc treatments was measured at 1.27, 1.02, and 0.72 mg/g, respectively, in comparison to that of CK (*p* < 0.01), presenting obvious inhibition between 20.63% and 55%.

### 3.4. Effect of YXYO on Reactive Oxygen Species (ROS) Accumulation and Antioxidant-Related Enzymes in A. flavus

**Figure 2:**
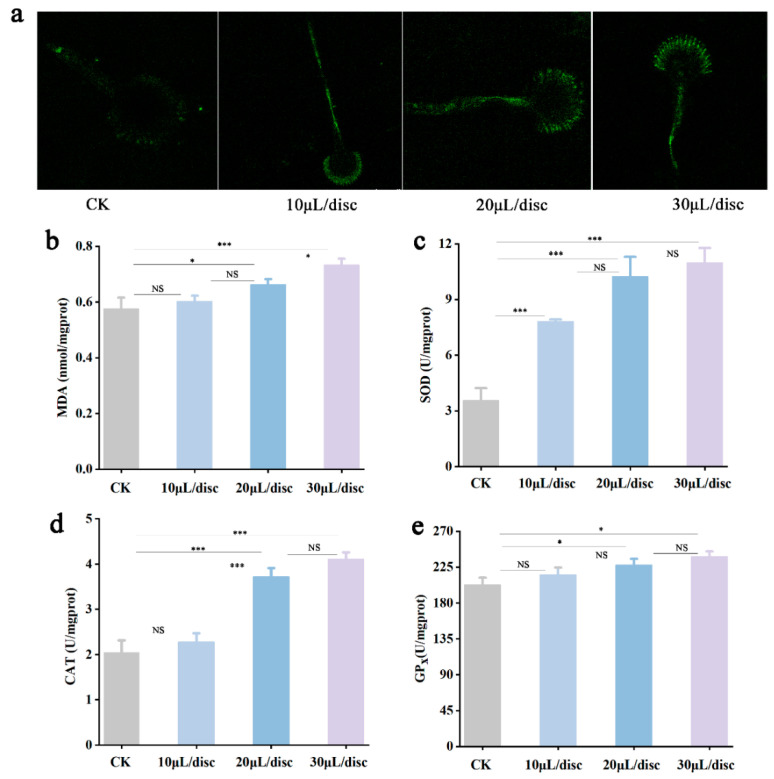
Effects of YXYO on oxidative impairment: (**a**) ROS accumulation, (**b**) MDA content, (**c**) SOD activity, (**d**) CAT activity, and (**e**) GPx activity. (*, *p* < 0.05; ***, *p* < 0.01; NS, no significant difference).

Cellular redox equilibrium is important for maintaining cell viability, and excess intracellular ROS will disrupt the state of redox equilibrium. ROS accumulation is considered an indicator of apoptosis, and the oxygen metabolism of cells is easily disturbed by ROS [32]. Excessive ROS can react readily with intracellular macro-molecules, leading to cell dysfunction and significant damage to cell structure [33]. To explore the mechanisms of YXYO against *A. flavus*, intracellular oxidative stress was investigated by examining ROS levels. As shown in Figure 2a, compared to the control, a notable enhancement in green fluorescence within the mycelia was observed following treatment with increasing concentrations of YXYO. This suggested that YXYO caused ROS accumulation in the mycelia. It is speculated that excess ROS may disturb the antioxidant defense system and cause different degrees of damage to proteins and lipids in cell membrane, as well as compromise the integrity of the cell membrane [1]. Malondialdehyde (MDA) is a breakdown product of lipid peroxidation, and it is often used to monitor lipid peroxidation due to oxidative damage [34], which can indirectly reflect the degree of tissue peroxidation damage [35]. The changes in the intracellular MDA content under different YXYO treatments are shown in Figure 2b. The MDA content in the 10 μL/disc, 20 μL/disc, and 30 μL/disc YXYO-treated groups was 0.60, 0.66, and 0.73 nmol/mgprot, respectively. The MDA content of the 20 μL/disc YXYO-treated group was significantly higher than that of the control group (0.57 nmol/mgprot). All these results suggest that YXYO may cause lipid peroxidation in *A. flavus* filament membranes, thus damaging the cell membrane.

When fungi are subjected to external pressure, the mold can effectively remove superoxide anions and other free radicals by activating antioxidant enzymes to promote the defense function. In order to explore the oxidative stress caused by YXYO in *A. flavus*, the activities of several antioxidant enzymes were measured. The effects on the antioxidant enzyme activities (superoxide dismutase (SOD), catalase (CAT), and glutathione peroxidase (GPx)) under different concentrations of YXYO treatment are shown in Figure 2c–e. The SOD, CAT, and GPx activities of *A. flavus* increased to varying degrees after treatment with 10, 20, and 30 μL/disc of YXYO. The antioxidant defense system serves as a crucial protective barrier against the oxidant damage induced by ROS. Among them, SOD can catalyze O_2_^−^ to molecular oxygen (O_2_) and H_2_O_2_; then, CAT can further catalyze the H_2_O_2_ to H_2_O, and GPx can use GSH as a reductant to catalyze H_2_O_2_ or organic hydroperoxides into water or the corresponding alcohols [36]. These results suggest that YXYO-triggered ROS accumulation activated antioxidative enzymes in the mycelia of *A. flavus*, resulting in the activation of the antioxidant defense system to preserve cellular homeostasis. This observation corroborates the research conducted by S. Das et al. [37], and P. P. Singh et al. [38]. Nonetheless, distinct essential oils exhibit diverse impacts on the antioxidant defense mechanism. For example, as the concentrations of the combined essential oils from *Ocimum* spp. increased, there was a corresponding gradual decline in the activity of SOD [39]. The findings of L. Liang et al. [40], corroborated this result, revealing that the activities of SOD, peroxidase (POD), and CAT demonstrated significant reductions following treatment with estragole. It is hypothesized that the excessive accumulation of ROS leads to a state of oxidative stress in fungi that surpasses the protective capabilities of antioxidant enzymes, consequently intensifying cellular damage.

### 3.5. Effect of YXYO on Energy Metabolism

**Figure 3:**
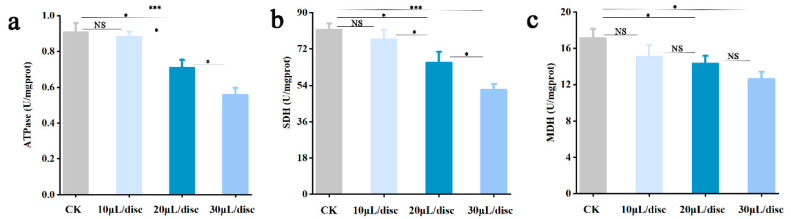
Effects of YXYO on energy metabolism: (**a**) ATPase activity, (**b**) SDH activity, and (**c**) MDH activity. (*, *p* < 0.05; ***, *p* < 0.01; NS, no significant difference).

Mitochondria, key organelles in cells of higher organisms, can produce ATP through the energy metabolism pathway, which is necessary for cellular physiological activity. Therefore, the inactivation mechanism of YXYO against *A. flavus* spores was further studied via energy metabolism. The localization of mitochondrial ATPase on the mitochondrial membrane is intricately associated with the energy metabolism of cells. As displayed in Figure 3a, the activity of ATPase was gradually reduced with increased YXYO treatment concentrations. Previous studies have similarly also found that some fungicides could inhibit fungal growth by decreasing ATPase activity [41,42].

The selected enzymes for the experiment were succinate dehydrogenase (SDH) and malate dehydrogenase (MDH), both of which are key enzymes in the mitochondrial tricarboxylic acid (TCA) cycle for ATP synthesis. SDH is one of the hubs connecting oxidative phosphorylation and electron transport, which provides electrons for the respiratory chain in eukaryotic mitochondria and prokaryotic cells. MDH catalyzes the reversible conversion of malic acid to generate oxaloacetic acid, which plays an important role in various cellular processes [43]. The activities of SDH and MDH in *A. flavus* mycelia were decreased in response to increasing concentrations of YXYO. As shown in Figure 3b, the activity of SDH showed a reduction of between 5.88% and 45.60% after treatment with YXYO. Similarly, MDH activity exhibited a decrease ranging from 12.05% to 31.45% compared with CK (Figure 3c). Our results corroborate the findings of C. Sarathambal et al. [44], indicating that *Pimenta dioica* leaf EO could inhibit the MDH activity with a dose-dependent mode. These results suggest that YXYO might hinder ATP production via the tricarboxylic acid cycle in *A. flavus* mycelia. This disruption in energy metabolism leads to mitochondrial dysfunction, which ultimately contributes to the inactivation of the fungi by YXYO.

### 3.6. Transcriptomic Analysis

**Figure 4:**
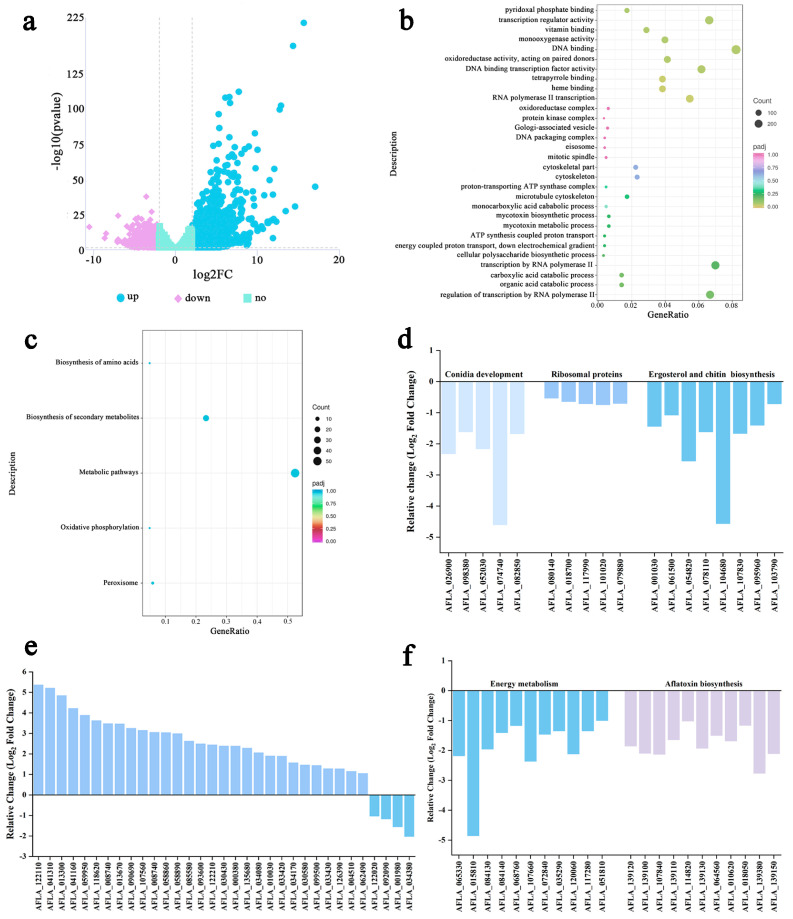
Transcriptome analysis of *A. flavus* treated with YXYO. (**a**) Volcano map of differential gene expression, (**b**) GO analysis, (**c**) KEGG analysis, (**d**) differentially expressed genes related to fungal development and cell barrier, (**e**) differentially expressed genes related to oxidative stress, and (**f**) differentially expressed genes related to energy metabolism and aflatoxin biosynthesis.

In order to explore the latent molecular mechanism response to YXYO in *A. flavus*, a transcriptomic analysis was performed for mycelial treatment with 0 (control group, CK) and 20 μL/disc. Differentially expressed genes (DEGs) were detected with a *p* value ≤ 0.05 and |log2 (fold change)| > 1. In total, 2870 DEGs were identified in the comparison groups, including 1957 upregulated genes and 913 downregulated genes (Figure 4a), which showed that the expression levels of genes in *A. flavus* were strongly influenced during the inhibition of mycelial growth by 20 μL YXYO. To analyze the functions of DEGs, the GO functional and KEGG metabolic pathways were enriched. It was showed that 83 GO terms were enriched in the comparison groups, among which the mycotoxin metabolic process, mycotoxin biosynthetic process, oxidoreductase activity, and monooxygenase activity were enriched (Figure 4b). Only the peroxisome, oxidative phosphorylation, metabolic pathways, biosynthesis of secondary metabolites, and biosynthesis of amino acids pathways were enriched by KEGG analysis (Figure 4c). The results showed that the function of DEGs was related to the regulation of fungal growth and secondary metabolism, which indicated that 20 μL YXYO could affect the growth of mycelia and the synthesis of secondary metabolites in *A. flavus*. This is consistent with the observed morphological changes.

#### 3.6.1. Analysis of the Genes Related to Fungal Development and Cell Barrier

The SEM images clearly showed abortive conidiophores and reduced conidia (Figure 1f). This is potent evidence of the YXYO inhibition of *A. flavus* development. Fungal differentiation and secondary metabolism are coordinated by many regulators in *Aspergillus*, among which the velvet regulators play a vital role [45]. As important members of the velvet family, VosA (AFLA_026900, developmental regulator VosA) governs spore viability and maturation, and WetA (AFLA_052030, developmental regulatory protein WetA) influences the synthesis of crucial conidial wall components at the late phase of conidiation, which makes the conidia impermeable and mature [46]. Transcriptome sequencing revealed that the VosA and WetA gene expression levels were clearly decreased in the YXYO treatment group (Figure 4d). In addition, some conidia-specific genes (AFLA_098380 and AFLA_074740) which contribute to the structural integrity and viability of the cell wall [47,48] were also downregulated (Figure 4d). Ribosomal proteins (RPs) are important for all types of cells to grow and survive [49]. Ribosomes serve as the cellular machinery responsible for protein synthesis, primarily functioning to translate genetic information into amino acid sequences [50]. *Cymbopogon citratus* EO nanoemulsion influenced the expression of genetic material by modulating the expression of genes associated with ribosomal structure and function [51]. It was found through transcriptome analysis that the expression levels of five genes (AFLA_080140 (60S ribosomal protein L12), AFLA_018700 (60S ribosomal protein L5), AFLA_117990 (ribosomal protein S5), AFLA_101020 (40S ribosomal protein S4), AFLA_079880 (60S ribosomal protein L22)) related to ribosomes decreased after YXYO treatment.

The plasma membrane plays a crucial role in maintaining homeostasis, exchanging materials, and the transduction of information [52]. Ergosterol is a key constituent of the fungal membrane. The transcriptional levels of several genes were downregulated after YXYO treatment, for example, AFLA_054820 (sterol delta 5,6-desaturase Erg3), AFLA_001030 (lanosterol synthase), AFLA_078110 (C-14 sterol reductase), and AFLA_061500 (squalene monooxygenase Erg1). Comparable effects were observed with linalool, a biogenic volatile organic compound derived from various plants, which demonstrated antifungal properties by compromising the integrity of cell membrane structures [53]. Certainly, there exists another mechanism of membrane disruption involving 2-ketobutyrate, which impairs membrane integrity by inhibiting sphingolipid synthesis through the downregulation of related metabolic genes. 2-ketobutyric acid inhibits sphingolipid synthesis by downregulating the expression of genes associated with sphingolipid metabolism [54]. Chitin is a major constituent of the matrix of many fungal walls and determines the integrity of the cell wall [55]. In the current study, the genes AFLA_104680 (class V chitinase ChiB1), AFLA_095960 (chitin synthase D), AFLA_107830 (brain chitinase and chia), and AFLA_103790 (chitin synthase activator Chs3), related to chitin synthesis, were significantly downregulated, suggesting that the chitin content in *A. flavus* might decrease after treatment with YXYO. Some genes related to chitin synthesis were also downregulated in *A. flavus* after treatment with honokiol [48]. Therefore, the findings indicated that YXYO disrupts the cell wall and cell membrane of *A. flavus*, resulting in functional hindrance. These results confirmed that YXYO could suppress the critical gene expressions of cell barrier formation and conidial development and further inhibited AF production.

#### 3.6.2. Analysis of the Genes Related to Oxidative Stress

Antifungal-agent-induced oxidative stress is intricate and causes irreversible damage to fungi. High ROS concentrations promote the release of various apoptotic factors and induce apoptosis, thereby inhibiting the growth of *Aspergillus* mycelia. As shown in Figure 4e, the expression levels of AFLA_122110 (Cat2) and AFLA_090690 (Cat1), which encode catalases, were upregulated. Additionally, the expression levels of AFLA_099000 (SOD1) and AFLA_068080, which encode Cu and Zn superoxide dismutase, were significantly increased in the treated group, consistent with the results of biochemical detection. This result demonstrates that the homeostasis between the formation and elimination of ROS through enzymatic antioxidants was disrupted following treatment with EOs [48,56]. However, following treatment with *p*-anisaldehyde, there was an observed upregulation of SOD2 expression, whereas Cat2 expression was significantly inhibited [57]. Furthermore, o-vanillin may modulate the expression of antioxidant enzymes, specifically SODs and CAT, through the downregulation of bZIP transcription factor genes [58]. In addition, the expressions of AFLA_041310, AFLA_013300, AFLA_041160, AFLA_059950, AFLA_118620, AFLA_008740, AFLA_013670, AFLA_107560, AFLA_008740, AFLA_058860, AFLA_058890, AFLA_085580, AFLA_093600, and AFLA_122210, which encode oxidoreductase, were all upregulated, predicting that *A. flavus* reduces the oxidative damage from ROS by increasing the activity of antioxidant enzymes.

#### 3.6.3. Analysis of the Genes Related to Energy Metabolism

It was previously observed that energy metabolism was suppressed when *A. flavus* mycelia were subjected to YXYO. The TCA (tricarboxylic acid) cycle is one of the important energy metabolism pathways occurring in the mitochondria in fungi [59]. In the current study (Figure 4f), it was found that the expression levels of several genes associated with TCA were reduced, which included AFLA_065330, AFLA_117280 encoding malate dehydrogenase, AFLA_068760, AFLA_107660 encoding succinate dehydrogenase, AFLA_015810 encoding citrate synthase, AFLA_84130, AFLA_84140, and AFLA_035290 encoding ketoglutarate and pyruvate dehydrogenase. These results are consistent with the mentioned key enzyme activity, SDH and MDH. In addition, the expressions of some genes (AFLA_072840, AFLA_120060, AFLA_136740, AFLA_083640) related to energy metabolism were also suppressed to varying degrees. Furthermore, the energy metabolism of *A. flavus* may be disrupted via various mechanisms, particularly upon exposure to linalool, with notable alterations in gene expression related to mitochondrial membrane transport, the tricarboxylic acid cycle, oxidative phosphorylation, and ATP synthesis [53]. These results suggest that YXYO might disrupt the normal progression of the TCA cycle and further lower energy metabolism in *A. flavus* mycelia.

#### 3.6.4. Analysis of the Genes Related to Aflatoxin Biosynthesis

To evaluate the regulatory roles of YXYO in aflatoxin biosynthesis, the expression levels of genes that are involved in aflatoxin biosynthesis were analyzed. The transcriptional expression of the majority of genes in the AF cluster was significantly increased after YXYO treatment (Figure 4f). It is noticed that the initial-step genes of AF biosynthesis, such as AFLA_139380 (aflA), AFLA_139130 (aflB), AFLA_139120 (aflC), AFLA_139110 (aflD), and AFLA_139100 (aflE), were significant suppressed by YXYO, while the medium- and later-stage genes were less affected. Furthermore, the transcript levels of other genes related to aflatoxin biosynthesis, such as AFLA_107840, AFLA_064560, AFLA_010620, and AFLA_018050, were also suppressed. Therefore, YXYO inhibited aflatoxin production by reducing the transcriptional levels of the aforementioned toxin-related genes. However, certain volatile compounds, including cinnamaldehyde [52] and citral [60], exhibit a greater degree of downregulation in the latter portion of the aflatoxin biosynthetic gene cluster, specifically in genes such as aflG, aflL, aflO, and aflQ. Additionally, there exist volatile compounds that influence transcriptional regulators specific to aflatoxin biosynthesis pathways, aflR and aflS [25,61]. Eugenol treatment suppressed nearly all AFB_1_ biosynthesis genes except one, accompanied by the downregulation of the regulatory complex formed by the two internal factors, aflR and aflS [62]. The various action targets of volatile organic compounds in *A. flavus* suggest that a strategic integration of these natural gaseous fungicides could facilitate the optimization of their individual dosages and mitigate the development of resistance in spoilage fungi.

### 3.7. Analysis of Volatile Compounds in YXYO by SPME-GC-MS

**Figure 5:**
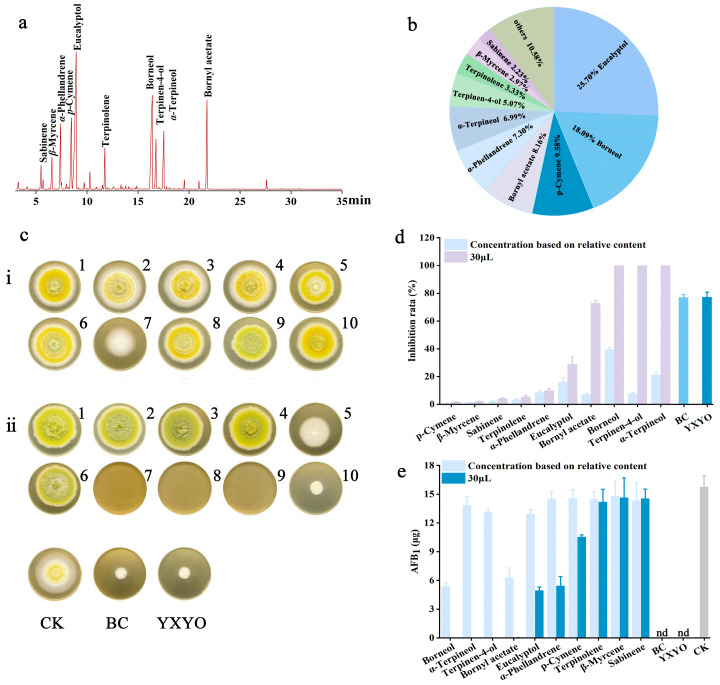
Volatile compounds and antifungal effect of YXYO. (**a**) GC-MS chromatogram of YXYO. (**b**) Related content of main compounds of YXYO. (**c**) Antifungal effects of major components and their blends in YXYO (the 10 compounds numbered from 1 to 10 are as follows: sabinene, *β*-myrcene, *α*-phellandrene, *p*-Cymene, eucalyptol, terpinolene, borneol, terpinen-4-ol, *α*-terpineol, and bornyl acetate): (**i**) the inhibitory effects of 10 individual components determined by their relative content in 30 μL YXYO; (**ii**) the inhibitory effects of 10 individual components at 30 μL/disc. (**d**) Inhibitory effects of YXYO on *A. flavus* growth. (**e**) Inhibitory effects of YXYO on AFB_1_ content. BC: blend of 10 major compounds determined by relative concentrations of 30 μL YXYO. (nd, not detected).

The effectiveness of the antimicrobial properties of essential oils is related to the active components present within them. This effect may arise from a single component or from a synergistic combination of multi-ingredients. The fumigation method was used in the antifungal test, necessitating the analysis of the volatile compounds in YXYO through SPME-GC-MS (Figure 5a). Factors affecting the results, such as the volatile oil extraction method, SPME fiber type, separation conditions, etc., were optimized in the pre-experiment. Under the optimized conditions, a total of 28 main compounds of YXYO were identified based on MS, RIs, and reference compounds. The names, molecular formulas, and relative abundances of these compounds are listed in Table S1. Among these compounds, the most abundant were sabinene (2.23%), *β*-myrcene (2.97%), *α*-phellandrene (7.30%), *p*-cymene (9.58%), eucalyptol (25.70%), terpinolene (3.33%), borneol (18.09%), terpinen-4-ol (5.07%), *α*-terpineol (6.99%), and bornyl acetate (8.16%). Collectively, these 10 compounds accounted for 89.42% of the overall composition of YXYO (Figure 5b). The chemical compounds of YXYO were consistent with previous research, which identified eucalyptol (21.67%) and borneol (17.37%) as the predominant constituents of the essential oil [63], although the quantities of the chemical components varied due to different abiotic and biotic factors.

To identify the active components, an investigation was conducted on the antifungal properties of these 10 primary components (Figure 5c). As illustrated in Figure 5d, there was no significant difference between the antifungal effects of the blend of these 10 major volatile components (76.89%) and YXYO (77.11%). This result suggests that these 10 components are the active ingredients for YXYO to exert antifungal effects. However, the antifungal effect of each ingredient was tested individually based on its relative concentration within 30 μL of YYXO, and the inhibition rates were found to range from 0.65% (*p*-cymene) to 39.33% (borneol). The effects of different treatments on aflatoxin production were also investigated using AFB_1_ as an indicator. As can be seen from Figure 5e, no AFB_1_ was detected after treatment with the blend and YXYO. Among these 10 components, borneol (5.34 μg/disc) and bornyl acetate (6.28 μg/disc) significantly inhibited the production of AFB_1_. In contrast, the levels of AFB_1_ after treatment with the remaining components were in the range of 12.90~15.48 μg/disc, which did not differ significantly from the control (15.73 μg/disc).

In addition, the effects of these 10 components on the fungal growth and aflatoxin accumulation of *A. flavus* at the same concentration were investigated. From Figure 5d, it can be seen that borneol, terpinen-4-ol, and *α*-terpineol inhibited *A. flavus* by 100%, followed by bornyl acetate (72.76%), and the remaining compounds demonstrated inhibition rates ranging from 1.58% to 28.80%. While eucalyptol constitutes the primary component of YXYO, its efficacy in inhibiting fungal growth and AFB_1_ is inferior to that of YXYO. This discrepancy may be attributed to the potential three types of interactions among the various constituents of the essential oil as a whole: additive, antagonistic, or synergistic effects [64]. AFB_1_ was not detected after treatment with borneol, α-terpineol, and terpinen-4-ol. Additionally, bornyl acetate demonstrated an inhibition rate approaching 100% against AFB_1_. Eucalyptol, *α*-phellandrene, and *p*-cymene also showed significant inhibitory effects on AFB_1_. It is noteworthy that the antifungal effect and the inhibition of aflatoxin production of some ingredients (*α*-phellandrene, *p*-cymene, etc.) varied considerably, suggesting the different antifungal and antiaflatoxin mechanisms associated with this class of compounds.

Although many studies have demonstrated the significant bioactivity of EOs derived from cinnamon bark, the comprehensive advantages—including a faster production cycle, higher yield efficiency, and notable antifungal properties—provide a strong justification for the focus on leaf-derived EO in this study. EOs extracted from the bark of *Cinnamomum jensenianum* Hand.-Mazz and *Cinnamomum cassia* have demonstrated antifungal and antiaflatoxin production effects against *A. flavus* [65,66]. Additionally, the bark of *Cinnamomum burmannii* has exhibited significant antimicrobial properties against various pathogens, including *Candida albicans*, *Staphylococcus aureus*, and *Salmonella anatum* [67,68]. Its primary active component, cinnamaldehyde, is recognized as an effective natural preservative against *A. flavus* [52]. However, this study focuses on leaf-derived EOs for several compelling reasons. The selection of leaf EO was primarily based on its superior economic feasibility and sustainable production potential. Notably, the maximum EO yield obtained from *Cinnamomum burmannii* leaves reached 1.36 ± 0.31 wt% after only 5 years of growth, in contrast to the bark and branches which required a significantly longer maturation period of 12 years to achieve comparable production levels [69]. This substantial difference in production efficiency makes leaf EO a more viable option for commercial applications. Furthermore, the primary components of YXYO, such as borneol and eucalyptol, have demonstrated remarkable biological activity in this study, showing significant efficacy in inhibiting both the growth of *A*. *flavus* and AFB_1_ production.

### 3.8. In Vivo Effects of YXYO on Peanut Infection of A. flavus

**Figure 6:**
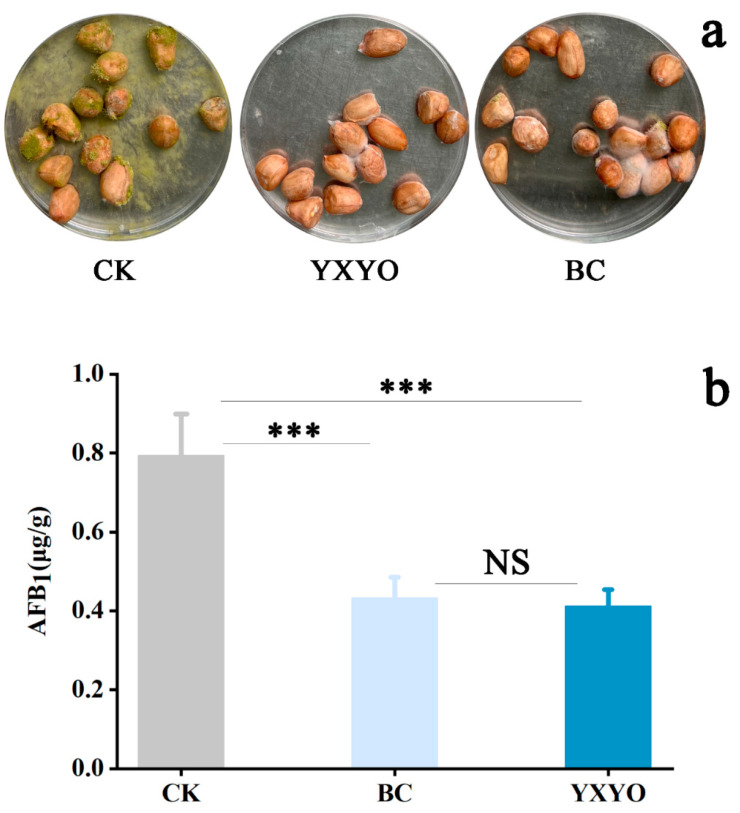
Inhibitory effects of YXYO on (**a**) *A. flavus* growth and (**b**) AFB_1_ accumulation in peanuts. (***, *p* < 0.01; NS, no significant difference).

The above results indicate that YXYO exhibits an antifungal effect on the mycelial growth and biosynthesis of aflatoxin of *A. flavus*. Finally, the antifungal efficacy of YXYO and a blend of 10 main compounds on peanuts was evaluated. As shown in Figure 6a, *A. flavus* spores germinated and grew normally on peanut butter within 7 d. After treatment with 30 μL/disc YXYO and the blend, AFB_1_ decreased by 48.11% and 45.56%, respectively (Figure 6b). These results clearly demonstrate that YXYO could be a potential effective antifungal agent to control *A. flavus* in peanuts. Nonetheless, the chemical instability, volatile nature, hydrophobic nature, and organoleptic effect of YXYO in food systems continue to hinder its large-scale application. To address these limitations, encapsulation systems, such as nanoparticles and various packaging techniques, represent a highly effective strategy for maintaining the biological activities of EOs while reducing their impact on the sensory attributes of food [70,71]. H. Tian et al. [72], found that unencapsulated citral, the main component of lemon eucalyptus oil, decreased by 91.78% after 12 days of storage; however, when encapsulated in solid lipid nanoparticles, it was reduced to only 33%. Moreover, it is crucial to acknowledge that an effective encapsulation technique can significantly enhance the antimicrobial efficacy of EOs [73,74]. Future research will aim to develop effective strategies to overcome the existing challenges of unencapsulated EOs and bioactive compounds utilized as environmentally friendly food preservatives [75,76]. In addition, the safety profile of YXYO requires careful consideration in practical applications. While there is a lack of studies substantiating the safety of YXYO, M. Ahmad et al. [77], indicated that the methanol extract of *Cinnamomum burmannii* exhibited no toxic effects in both acute (14-day, single-dose) and subchronic (28-day, repeated-dose) toxicity assessments conducted on Sprague Dawley rats. This may suggest that YXYO, as a related natural product, is likely to be safe as well [78].

## 4. Conclusions

*Cinnamomum burmannii* leaf essential oil exhibited a significant inhibitory effect on the growth of *Aspergillus flavus* and a reduction in AF production. The inhibition mechanisms of YXYO against *A. flavus* growth and AF production were explored. The results showed that YXYO inhibited mycelial growth, disrupted cell membrane integrity, and heightened oxidative damage. Biochemical experiments further confirmed that some key enzymes related to oxidative stress and energy metabolism were significantly affected by YXYO. Consistently, the transcriptomics results showed that the YXYO treatment remarkably regulated the expression of some genes related to conidial development, ergosterol synthesis, oxidative stress, energy metabolism, and aflatoxin biosynthesis, revealing the molecular mechanisms of its inhibitory effects. In addition, the antifungal active ingredients in YXYO were identified and substantiated. The antifungal effects of YXYO and a blend of its active compounds have been confirmed in peanut seeds. In conclusion, YXYO presents promising potential as a biofumigant for the prevention and management of *A. flavus* and aflatoxin contamination during the storage of agricultural products.

## Data Availability

The original contributions presented in this study are included in the article/Supplementary Materials. Further inquiries can be directed to the corresponding authors.

## Supplementary Materials

The following supporting information can be downloaded at: https://www.mdpi.com/article/10.3390/foods14040682/s1, Table S1. Main compounds of YXYO analyzed by SPME-GC-MS.
