# Supplementary material for: Inhibition Mechanism of Cinnamomum burmannii Leaf Essential Oil Against Aspergillus flavus and Aflatoxins
Source: Foods. 2025 Feb 17;14(4):682. doi: 10.3390/foods14040682 (PMC11853908; doi:10.3390/foods14040682)
Supplement: Supplementary file 1 [file foods-14-00682-s001.zip › foods-3373507-supplementary.pdf]

Table S1. Main compounds of YXYO analyzed by SPME-GC-MS.

NO	R. T. (min)	Compounds	Molecular formula	R. I.	Pct Total (%)
1	3.259	*α*-Pinene	$C_{10}H_{16}$	933	0.69±0.03
2	4.137	Camphene	$C_{10}H_{16}$	948	0.28±0.04
3	5.491	Sabinene	$C_{10}H_{16}$	970	2.23±0.11
4	5.695	*β*-Pinene	$C_{10}H_{16}$	977	0.86±0.05
5	6.555	*β*-Myrcene	$C_{10}H_{16}$	990	2.97±0.15
6	7.399	*α*-Phellandrene	$C_{10}H_{16}$	1005	7.30±0.92
7	7.973	3-Carene	$C_{10}H_{16}$	1010	0.47±0.07
8	8.14	*α*-Terpinene	$C_{10}H_{14}$	1016	0.24±0.02
9	8.463	*p*-Cymene	$C_{10}H_{14}$	1022	9.58±1.12
10	8.915	Eucalyptol	$C_{10}H_{18}O$	1031	25.70±2.04
11	9.172	trans-*β*-Ocimene	$C_{10}H_{16}$	1037	0.12±0.01
12	9.725	cis-*β*-Terpineol	$C_{10}H_{18}O$	1043	0.48±0.06
13	10.279	*β*-Ocimene	$C_{10}H_{16}$	1045	1.31±0.14
14	11.52	*γ*-Terpinene	$C_{10}H_{16}$	1056	0.32±0.02
15	11.742	Terpinolene	$C_{10}H_{16}$	1090	3.33±0.56
16	12.608	β-Linalool	$C_{10}H_{18}O$	1100	0.33±0.02
17	13.331	Cyclohexane,2-ethenyl-1,1-dimethyl-3-methylene-	$C_{11}H_{18}$	1118	0.30±0.07
18	13.789	cis-2-*p*-Menthen-1-ol	$C_{10}H_{18}O$	1123	0.28±0.03
19	14.089	3-Butenoic acid, 3-methyl-, (3-methyl-3-butenyl) ester	$C_{10}H_{16}O_2$	1130	0.20±0.02
20	14.875	Camphor	$C_{10}H_{16}O$	1148	0.25±0.04
21	16.377	Borneol	$C_{10}H_{18}O$	1171	18.09±1.58
22	16.746	Terpinen-4-ol	$C_{10}H_{18}O$	1177	5.07±0.75
23	17.046	*p*-Cymen-8-ol	$C_{10}H_{14}O$	1181	0.28±0.05
24	17.516	*α*-Terpineol	$C_{10}H_{18}O$	1188	6.99±0.84
25	19.538	*β*-Citral	$C_{10}H_{16}O$	1240	0.72±0.06
26	20.968	*α*-Citral	$C_{10}H_{16}O$	1271	0.63±0.09
27	21.748	Bornyl acetate	$C_{12}H_{20}O_2$	1290	8.16±0.97
28	27.604	Caryophyllene	$C_{15}H_{24}$	1420	0.75±0.05

R. T. = retention time; R. I. = retention index.
